# Serum pro-metalloproteinase 9 is a predictor of length of ICU and hospital stay in patients with septic shock

**DOI:** 10.1186/cc12670

**Published:** 2013-06-19

**Authors:** NA Costa, AF Gonçalves, BPM Rafacho, AL Gut, PS Azevedo, LAM Zornoff, SAR Paiva, MF Minicucci

**Affiliations:** 1 Botucatu Medical School, UNESP, Rubião Júnior, Botucatu, SP, Brazil

## Introduction

The matrix metalloproteinases (MMPs) participate in fundamental processes, such as cell proliferation, differentiation, adhesion, migration, angiogenesis, apoptosis, and inflammation [1]. The increased expression of MMPs suggests that these proteases may influence the pathogenesis of endotoxemia in sepsis [2]. The objective of this study was to evaluate the serum activity of MMP-2 and MMP-9, length of hospital stay, length of ICU stay and mortality in patients with septic shock.

## Methods

This prospective study included all patients with septic shock on admission or during ICU stay, over the age of 18 years, admitted to the ICU from March to July 2012. Demographic information, clinical evaluation and blood samples were taken within the first 72 hours of the patient's admission or within 72 hours after septic shock diagnosis for laboratory analysis and MMP-2 and MMP-9 activity. The activity of MMPs was performed by zymography. The level of significance was set at 5%.

## Results

We evaluated 67 patients with a mean age of 56 ± 15 years, 66% male, the median length of ICU and hospital stay was 9 (4 to 15) and 16 (10 to 29) days, respectively, and the ICU mortality rate was 61%. Higher values of APACHE II, SOFA, lactate and urea, and lower values of albumin, length of ICU and hospital stay were associated with ICU mortality. In univariate analysis, serum activity of MMP-2 and MMP-9 were not associated with length of ICU, hospital stay and mortality in septic shock patients. However, in the regression model analysis when adjusted for sex, age, lactate and APACHE II, the activity of pro-MMP-9 was negatively associated with the length of ICU (coefficient: -0.016; *P *= 0.028) and hospital (coefficient: -0.015; *P *= 0.041) stay, but was not associated with ICU mortality (OR: 1.051; 95% CI: 0.950 to 1.163; *P *= 0.332).

## Conclusion

Serum activity of pro-MMP-9 was negatively associated with length of ICU and hospital stay in patients with septic shock.
